# Editorial: Influence of dietary factors, nutrients, and the gut-lung axis on respiratory health

**DOI:** 10.3389/fnut.2025.1556801

**Published:** 2025-02-11

**Authors:** Inês Brandão, Tomoko Suzuki, Naser A. Alsharairi

**Affiliations:** ^1^Centro de Apoio Tecnológico Agro-Alimentar (CATAA), Castelo Branco, Portugal; ^2^Centre for Functional Ecology, TERRA Associate Laboratory, Department of Life Sciences, University of Coimbra, Coimbra, Portugal; ^3^Department of General Internal Medicine, Saitama Medical University, Saitama, Japan; ^4^Heart, Mind and Body Research Group, Griffith University, Gold Coast, QLD, Australia

**Keywords:** respiratory health, gut-lung axis, gut microbiota, dietary and nutritional intervention, gut and lung immunity, lung inflammation, short-chain fatty acids (SCFAs), asthma and COPD

Dietary habits and nutrients are gaining increasing recognition as modifiable factors influencing respiratory health, with the gut microbiota playing a pivotal role as a mediator of these interactions.

The mechanisms linking diet with gut microbiota modulation, which may in turn alter lung inflammation, are still to be unraveled. The interplay between diet, gut microbiota, and respiratory health appears to revolve around the immune system, making immune interactions a central topic of discussion. This Research Topic aims to address the links between diet/nutrition and respiratory health, and the relationship between diet and the gut-lung axis.

Several contributions used NHANES data to explore how dietary intake and nutrition-related indicators affect respiratory outcomes in adults, investigating potential biomarkers for predicting disease progression and even mortality. Wen et al. found no association between protein intake and blood eosinophil counts (BEOC) in asthmatic patients. However, serum albumin levels showed an inverted U-shaped correlation with BEOC, suggesting that the nutritional status of patients may influence immune system changes and asthma regulation. Zhuang et al. found no association between dietary protein intake and mortality, but revealed that higher serum albumin levels were inversely associated with all-cause mortality. Interestingly, each unit increase in serum albumin (g/L) was linked to a 13% reduction in mortality risk. Serum albumin could serve as a reliable predictor of long-term outcomes in asthmatic patients, surpassing the influence of dietary protein intake alone. Likewise, Lu et al. examined the relationship between dietary protein intake and all-cause mortality in COPD patients. While a U-shaped trend was observed, with the lowest mortality risk at 90.3 g/day, no association between protein intake and mortality was found. Although adequate protein intake is critical for muscle maintenance, higher intake did not improve survival in COPD patients. Xu et al. investigated multiple nutrition-related indicators, including the Geriatric Nutritional Risk Index (GNRI), Advanced Lung Index (ALI), and Prognostic Nutritional Index (PNI), in relation to COPD risk and all-cause mortality. The study found that malnutrition, as indicated by low ALI and GNRI, and high CONUT scores, was linked to increased COPD incidence and mortality. Compared to other nutritional scores, ALI provides more effective predictive value for both risk and all-cause mortality. Jiang et al. found a correlation between higher Dietary Inflammatory Index (DII) scores and an increased risk of sarcopenia. Sarcopenia was linked to higher all-cause mortality in COPD patients, suggesting that dietary inflammation plays a critical role in disease progression and survival outcomes.

A review by Zhang et al. highlighted the gut-lung axis in asthma development, showing how gut microbiota (e.g., *Lactobacillus*) and short-chain fatty acids (SCFAs), modulate lung immunity by reducing inflammation and regulating immune responses. Early-life gut dysbiosis and microbial diversity are linked to asthma risk, while factors like rural living and diet shape microbiota resilience. The review calls for exploring interventions like probiotics, fecal transplants, dietary changes, and microbial engineering as strategies for asthma prevention and management.

Bezemer et al. investigated the effects of a symbiotic combination of *Bifidobacterium breve* and a prebiotic fiber mixture (short-chain galacto-oligosaccharides, long-chain fructo-oligosaccharides, low-viscosity pectin) on a preclinical mouse model for pulmonary neutrophilia. The synbiotic mixture improved lung resistance, pulmonary neutrophil-to-lymphocyte (NLR) ratio, and provided relief from pulmonary neutrophilia. Lipopolysaccharide (LPS) applied to the upper airways negatively impacted fecal SCFA when comparing to mice receiving PBS. Acetic acid production was increased in all unchallenged mice (PBS) which after LPS challenge was observed only in mice receiving the symbiotic mixture. Moderate and weak correlations were found between SCFAs and lung function or NLR. These results highlight the bidirectional gut-lung crosstalk, suggesting synbiotic-induced SCFAs beneficial role in lung health.

Holloman et al., explored the potential of Indole-3-carbinol (I3C), a compound found in cruciferous vegetables and used as a dietary supplement, to alleviate LPS-induced acute respiratory distress syndrome (ARDS) in mice. I3C reduced lung inflammation by decreasing the recruitment of CCR2+ monocytes, CXCR2+ neutrophils, and downregulated chemokines that are critical for immune cell trafficking, thereby reducing neutrophil-mediated tissue damage. The effects of I3C were mediated by aryl hydrocarbon receptor (AhR) activation, as mice lacking AhR in myeloid cells did not respond to treatment. These findings suggest that CCR2+ monocytes facilitate neutrophil recruitment during ARDS, and targeting AhR with dietary ligands like I3C could provide a novel therapeutic strategy for managing ARDS.

Schenzel et al. investigated the effects of a fiber-rich diet on exacerbations triggered by respiratory infections. β-hydroxybutyric acid levels were reduced in asthmatic children after exacerbations, potentially due to gut dysbiosis caused by respiratory infections. In a mouse model of asthma, the fiber-rich diet alleviated lung inflammation, reduced eosinophils expressing FcεRIα, suppressed GATA3+ Th2 cells, increased CD8+ effector memory T cells, and enhanced antiviral immunity. These findings suggest that dietary fiber and β-hydroxybutyric acid could help reduce airway inflammation, Th2-mediated responses, and eosinophil activation, offering potential as a supportive therapy for asthma, especially during exacerbations.

Shi et al. examined serum GDF15 in patients with acute exacerbations of COPD (AECOPD). Serum GDF15 levels in patients with malnutrition and AECOPD were higher than those in patients without malnutrition, whilst the serum albumin levels were lower than those in patients without malnutrition. Serum GDF15 levels were an independent risk factor for malnutrition in patients with AECOPD. The combination of GDF15 and serum albumin levels revealed a higher predictive ability for malnutrition in patients with AECOPD.

Overall, these studies highlighted how dietary factors influence the gut-lung axis and how this translates in respiratory health. Key findings include the role of serum albumin and serum GDF15, in managing respiratory diseases. These biomarkers are particularly significant in identifying malnutrition, a critical factor that exacerbates disease progression and increases mortality risk, however there is need for further robust evidence to support their clinical application. Pro-inflammatory diets, linked to sarcopenia, exacerbate COPD mortality, while interventions like fiber-rich diets, probiotics, and bioactive compounds (e.g., I3C) showed promise in reducing lung inflammation and enhancing immunity. Let's not forget the major influence of SCFA in mediating these effects. These findings underscore the importance of dietary and microbiota-focused strategies in managing and preventing respiratory diseases.

